# Robot-assisted walking training for individuals with Parkinson’s disease: a pilot randomized controlled trial

**DOI:** 10.1186/1471-2377-13-50

**Published:** 2013-05-24

**Authors:** Patrizio Sale, Maria Francesca De Pandis, Le Pera Domenica, Ivan Sova, Veronica Cimolin, Andrea Ancillao, Giorgio Albertini, Manuela Galli, Fabrizio Stocchi, Marco Franceschini

**Affiliations:** 1 IRCCS “San Raffaele Pisana”, Via della Pisana 235, Rome 00163, Italy; 2 “San Raffaele Cassino” Institute, Tosinvest Sanità SpA, Rome, Italy; 3 Dipartimento di Bioingegneria, Politecnico di Milano, Milan, Italy

**Keywords:** Parkinson’s disease, Gait analysis, Lower limb, Robot

## Abstract

**Background:**

Over the last years, the introduction of robotic technologies into Parkinson’s disease rehabilitation settings has progressed from concept to reality. However, the benefit of robotic training remains elusive. This pilot randomized controlled observer trial is aimed at investigating the feasibility, the effectiveness and the efficacy of new end-effector robot training in people with mild Parkinson’s disease.

**Methods:**

*Design*. Pilot randomized controlled trial.

*Setting*. Robot assisted gait training (EG) compared to treadmill training (CG).

*Participants*. Twenty cognitively intact participants with mild Parkinson’s disease and gait disturbance.

*Interventions*. The EG underwent a rehabilitation programme of robot assisted walking for 40 minutes, 5 times a week for 4 weeks. The CG received a treadmill training programme for 40 minutes, 5 times a week for 4 weeks.

*Main outcome measures*. The outcome measure of efficacy was recorded by gait analysis laboratory. The assessments were performed at the beginning (T0) and at the end of the treatment (T1). The main outcome was the change in velocity. The feasibility of the intervention was assessed by recording exercise adherence and acceptability by specific test.

**Results:**

Robot training was feasible, acceptable, safe, and the participants completed 100% of the prescribed training sessions. A statistically significant improvement in gait index was found in favour of the EG (T0 versus T1). In particular, the statistical analysis of primary outcome (gait speed) using the Friedman test showed statistically significant improvements for the EG (p = 0,0195). The statistical analysis performed by Friedman test of Step length left (p = 0,0195) and right (p = 0,0195) and Stride length left (p = 0,0078) and right (p = 0,0195) showed a significant statistical gain. No statistically significant improvements on the CG were found.

**Conclusions:**

Robot training is a feasible and safe form of rehabilitative exercise for cognitively intact people with mild PD. This original approach can contribute to increase a short time lower limb motor recovery in idiopathic PD patients. The focus on the gait recovery is a further characteristic that makes this research relevant to clinical practice. On the whole, the simplicity of treatment, the lack of side effects, and the positive results from patients support the recommendation to extend the use of this treatment. Further investigation regarding the long-time effectiveness of robot training is warranted.

**Trial registration:**

ClinicalTrials.gov NCT01668407

## Background

The effectiveness of non-pharmacological treatment on gait impairment in Parkinson’s Disease (PD), such as exercises [1], and physiotherapy in particular [2-4], has been demonstrated. The goal of physiotherapy treatment aims at enabling people with PD to maintain their maximum level of mobility, activity and independence through monitoring their condition and targeting the appropriate treatment [5]. Several systematic reviews and clinical studies have shown that physical therapy can contribute to minimize the disabling effects of motor and sensory impairments, enhancing participation in societal roles and quality of life. In the last years, electromechanical devices such as treadmill training have also been used in PD patients. In particular, Mehrholz and colleagues have conducted a systematic Cochrane study to assess the effectiveness and the acceptability of treadmill training in the treatment of gait disorders for patients with PD [6]. Recently, a new Cochrane analysis showed that, in a high number of people, there were some improvements in all walking outcomes after physiotherapy intervention, but these improvements were only significant for walking speed, walking endurance and step length [5]. In the last ten years robotic assisted devices have been used for gait training in neurological disorder such as stroke, spinal cord injury and multiple sclerosis, with good results on gait recovery [7-14]. Until now only 3 studies have been conducted to assess the effects of exoskeleton or end effector robot-assisted training in PD patients, with some interesting preliminary results [15-17]. Our pilot randomized controlled trial (RCT) is aimed at investigating the feasibility and the effects on the walking performance of the new end-effector robotic rehabilitation locomotion training in 10 patients with mild PD, comparing them to 10 patients with mild PD that underwent a training treatment with treadmill. The purpose was to highlight the short time modification induced by an experimental treatment and to analyse the change on principal gait indexes.

## Methods

### Participants

This study was a pilot Randomized Controlled Trial (RCT). We recruited idiopathic PD patients from rehabilitation centres. They had been on stable doses of Parkinson’s medications for at least 4 weeks prior to study onset, and showed an endurance sufficient to keep an upright position, assisted or unassisted, for at least 20 minutes. A preliminary medical examination included a physical and a neurological test, and a gait analysis. The inclusion criteria for all groups were: (a) diagnosis of idiopathic PD by UK Brain Bank criteria, without any other significant neurological or orthopedic problems; (b) age between 18 and 90 years old; (c) capability to walk, unassisted or with little assistance, for 25 feet. The following exclusion criteria were identified: (d) inability to understand instructions required by the study (Informed Consent Test of Comprehension); (e) primarily wheelchair bound; (f) chronic and ongoing alcohol or drug abuse, active depression, anxiety or psychosis that might have interfered with the use of the equipment or testing; (g) diagnosis of atypical parkinsonian syndrome; (h) implantation of deep brain stimulation.

### Procedures

After providing written informed consent, the twenty patients were divided into two groups randomly: Experimental Group (EG) and Control Group (CG). The random allocation to treatment was concealed and based upon a custom computerized system, using a purpose-built software. In order to allow a balanced subject allocation into EG and CG groups, the Lehemer algorithm was applied. Therapists were assigned to each group of patients randomly. Blinded assessors conducted clinical assessments at the beginning (T0) and at the end of the treatment (T1).

### Measures

#### Clinical assessments

Trained professionals, who were not involved in the research treatment and blind to patients’ group allocation, performed all instrumental and clinical assessments. All outcome assessments were collected in ON phase one hour and half after the oral assumption of the usual dose of levodopa. Clinical and instrumental outcomes were performed using valid and reliable tools for PD. They included: Hoehn and Yahr scale (HY) [18], Unified Parkinson’s Disease Rating Scale (UPDRS) [19], and multifactorial 3D Gait Analysis (3D-GA) (ELITE2002, BTS, Italy).

#### *3D Gait analysis*

The 3D-Gait analysis (3D-GA) was conducted using the following equipment: a 12-camera optoelectronic system with passive markers (ELITE2002, BTS, Italy) to measure the kinematic of the movement; 2 force platforms (Kistler, CH), to obtain the kinetic data of the movement (i.e. ground reaction forces); 2 TV camera Video system (BTS, Italy) synchronized with the optoelectronic and force platform systems for video recording. To evaluate the kinematics of each body segment, markers were positioned as described by Davis and colleagues [20]. Subjects were asked to walk barefoot, at their own natural pace (self-selected and comfortable speed), along a 10 meter walkway where the two force platforms were placed. At least seven trials were collected for each subject in order to ensure the consistency of the data. All graphs obtained from GA were normalized as % of gait cycle, and kinetic data were normalized for individual body weight. In order to quantify the gait pattern of participants involved in this study, a specific software (Smartanalyser, BTS, Italy) enabled the calculations of some indices (time/distance parameters, angles joint values in specific gait cycle instant, peak values in ankle power graph) starting from those data.

#### *Primary and secondary outcomes*

A primary outcome was the gait velocity assessed by mean velocity (m/sec), which measured the rate of change of position, recorded in meters per second [21]. The other outcomes were: the cadence (step/min) that measured the number of steps taken in a given period of time, which was then converted into the number of steps taken per minute [21], the step length (mm) that measured the average distance (in meters) between two successive placements of the same foot [22], the stride length (mm) that measured the average distance (in meters) between two successive placements of the same foot [22], the step width (mm) that measured medio-lateral distance between the two feet during double support, the stance time (% stride) that measured the duration of the stance phase, the swing time (% stride) that measured the duration of the swing phase and the double support (% stride) that measured the duration of double support.

#### *Therapeutic intervention*

Patients underwent a cycle of out-patients rehabilitation treatment, consisting of at least a daily 3-hour cycle, divided as follows: 45 minutes of treatment for lower limb either with robot device or with treadmill, according to the randomization; a treatment of occupational therapy for the upper limb, including both dexterity and neuropsychological treatment, according to individually tailored exercise scheduling. The whole therapy was performed under the supervision of a physiotherapist.

##### Experimental group (EG

Each subject was asked to perform 20 sessions (5 days a week for 4 weeks) of robot assisted gait training, using the commercially available end effector system machines G-EO system device (Reha Technology AG; Olten, Switzerland).

The engineering characteristic of G-EO robot is based on end effector device with BWS and a foot plates placed on a double crank and a rocker gear system, and with 3 DoF each, which allows the control of the length and the height of the steps. The foot plate angles can be used to simulate a real over-ground high repetitive walk [23]. The trajectories of the foot plates and the vertical and horizontal movements of the centre of mass are fully programmable, enabling wheelchair-bound subjects not only for the repetitive practice of simulated floor walking, but also to climb up and down the stairs. Heart rate and blood pressure were monitored at the beginning and at the end of each session. During the training, the therapist followed the treatment standing in front of the patient, to help if necessary. The parameters of the treatment were noted for each session, and the steps taken during the simulated walking were converted into the distance covered, based on the step length previously chosen [24].

The practice included a robot-assisted walking therapy, at variable speeds, for 45 minutes, with a partial body weight support (BWS). All participants started with 30-40% of BWS and an initial speed of 1.5 km/h; afterwards, speed was increased to a range of 2.2 to 2.5 km/h maximum and initial BWS was decreased. After 45 minutes the session was stopped.

##### Control group

Each subject received 20 sessions (5 days a week for 4 weeks) of treadmill rehabilitation treatment. All subjects were asked by the therapist to perform a treadmill training treatment, at their best convenience, for 45 minutes, according to a protocol setting. The patients received video feedback to improve the gait quality. The Gait Trainer 3, equipped with Visual Biofeedback Screen, provided the necessary stimulus for retraining neural pathways, thus improving the patient’s gait pattern and assessing the patient’s ambulation progress The biofeedback parameters could be set by the therapist according to patient’s characteristic (i.e. impairment) and to the desired goals (i.e. improvement in velocity and/or step length and/or cadence). In particular, the patients could follow a graphical representation of the foot, on the treadmill screen, having a visual feedback of their performance simultaneously.

Heart rate and blood pressure were monitored at the beginning and at the end of each session. During the training sessions, the therapist followed the treatment standing on one side of the patient. The treatment parameters were noted for each session.

In both groups, subjects who did not retrieve sessions and interrupted the treatment for more than 3 consecutive days were excluded from the study.

### Statistical analysis

All the previously defined parameters were computed for each participant. Mean values and standard deviations of all indexes were calculated for each group. The Kolomogorov–Smirnov tests were used to verify if the parameters were normally distributed. As this was not the case, we used Wilcoxon’s tests in order to detect significant change between data at baseline (T0) and endpoint (T1). The T0 and T1 data of all patients and CG were compared with Mann–Whitney U tests. Statistical significance was set at p < 0.05. The Mann–Whitney test was used to compare median scores between groups.

### Ethical aspects

This study was performed in accordance with the Declaration of Helsinki and was approved by the ethics committees of IRCCS San Raffaele Pisana. Informed consent was obtained from all subjects enrolled in this study.

## Results

**Figure 1:**
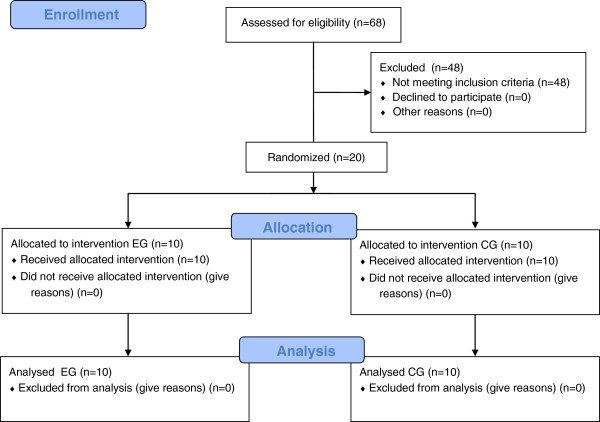
CONSORT 2010 flow diagram.

We screened 68 patients, 20 of whom satisfied the inclusion criteria and were randomly assigned to the groups as follows: 10 to the robot-assisted therapy (EG), 10 to the intensive therapy (CG) (Figure 1). The distribution of the study subjects (N = 20) by age, gender, and main clinical and demographical characteristics did not show significant difference between the EG and the CG (Table 1). Within each group, no dropouts were recorded during the treatment and all subjects fulfilled the protocol (compliant subjects: N = 20). The Mann–Whitney test showed no statistical significant differences at T0 between the two groups for age, height, weight, mean velocity, cadence, step width, step length, stride length, stance time, swing time, double support, stride time. Table 2 summarizes the observed mean ± standard deviation and the p value for all tests (T0 versus T1), as measured on the compliant subjects at T0 (N = 20), T1 (N = 20) (Table 2). The statistical analysis of primary outcome (gait speed), which used the Friedman test, showed statistically significant improvements for the EG (p = 0,0195). The statistical analysis performed by Friedman test of Step length left (p = 0,0195) and right (p = 0,0195) and Stride length left (p = 0,0078) and right (p = 0,0195) showed a significant statistical gain. No change in stance and swing time phase left and right in EG were found. No statistically significant improvements on the CG were found. The inter-group statistical analysis of the gain of all parameter did not show statistically significant improvements.

**Table 1:** Distribution of the study participants by age, gender, height, disease duration and other clinical characteristics

	**Experimental-group**		**Control-group**	
Subjects (M/F)	10 (6/4)		10 (5/5)	
Height (m)	1.59 ±0.10		1.60 ±0.12	
Age (years)	70.27 ±9.81		68.42 ± 9.41	
Disease duration (years)	8.41 ±4.99		8.72 ±4.74	
Hoehn & Yahr stage (range)	2.5 – 3.5		2.5 - 3.5	
	T0 session	T1 session	T0 session	T1 session
UPDRS III score	53.57 ±14.74	40.45 ± 7.88 p = 0.0027	56.17 ±13.86	40.25 ±8.21 p = 0.0055

**Table 2:** Results of observed means ± Standard Deviation (SD) of gait analysis spatio-temporal parameters and significant P value of Control Group (CG), Experimental Group (EG) and Normal subject Group (NG) at T0 and T1

	**CG-group**					**EX-group**					**NG-group**
	**Mean**	**S.D**	**Mean**	**S.D.**	**P value**	**Mean**	**S.D**	**Mean**	**S.D.**	**P value**	
	**TO**		**T1**			**TO**		**T1**			
Mean velocity [m/sec]	0,5711	0,3068	0,7022	0,2721	0,1016	0,58	0,1679	0,7644	0,202	0,0195*	1.2 ±0.01
Cadence [step/min]	88,67	12,81	93,11	17,87	0,1953	98,89	10,29	105,3	9,421	NS	113.8 ±4.3
Step width [mm]	175,6	21,28	185,6	25,06	0,1875	131,8	29,38	134,9	15,85	NS	115.5 ± 25.9
Step length [mm] DX	402,2	137,6	427,8	127,6	0,7422	337,9	89,76	405	82,92	0,0195*	
Step length [mm] SX	372,2	122	441,1	82,68	0,0391*	373,7	103,7	448,4	114,4	0,0195*	
Stride length [mm] DX	867,8	326,6	953,3	218,5	0,5703	694,8	195,8	892,2	239,1	0,0078*	
Stride length [mm] SX	860	302,5	946,7	215,2	0,4258	714,6	187,7	853,7	186,5	0,0195*	
Stance time [% stride] DX	63,11	5,395	64,83	4,886	0,3008	63,22	3,193	63,33	3,969	NS	58 - 61
Swing time [% stride] DX	36,94	5,294	35,17	4,886	0,2891	36,89	3,219	36,67	3,969	NS	
Stance time [% stride] SX	66,89	2,804	64,31	2,592	0,082	64	3,873	61,44	4,475	NS	57 - 61
Swing time [% stride] SX	33,26	2,788	35,69	2,592	0,1289	36,11	3,919	38,56	4,475	NS	
Double supp. [% stride] DX	15,41	4,127	14,7	3,685		14,44	4,851	13,33	5,099		
Double supp. [% stride] SX	15,58	3,662	14,72	3,73		12,22	2,167	12,22	4,265		

* indicate statistically significant results.

## Discussion

The management of PD has been traditionally centred on drug therapy, with levodopa seen as the “gold standard” treatment. However, there has been recently an increasing support for the inclusion of rehabilitation therapies as an adjuvant to pharmacological and neurosurgical treatment [25,26]. Furthermore, the guidelines published by the National Collaborating Centre for Chronic Conditions recommended that physiotherapy be made available throughout all stages of the disease, raising the profile of the profession. Walking ability, though important for quality of life and participation in social and economic living, can be adversely affected by neurological disorders such as PD, spinal cord injury, stroke or traumatic brain injury. The ability to walk safely and independently at an acceptable speed, and therefore regaining a level of functional gait [27], is an important factor which enables patients to lead an autonomous and self-determined life. Various studies concerning the functional evaluation of subjects with PD are present in literature, but evidences concerning the outcomes of the quantitative gain, after treatments, are very little. The measurement of temporal and spatial features of gait is essential for the assessment of gait abnormalities and the quantitative evaluation of treatment outcomes [27]. Many studies about different neurological diseases showed that the robotic devices have been developed to relieve physical therapists of the strenuous and unergonomical burden of treadmill BWS (Body Weight Support) training [7-29]. For gait training, then, the repetitive walk in a natural gait, similar to over-ground gait, and with the correct proprioceptive and exteroceptive feedback, is of great importance. The first study about a robotic rehabilitation in PD patients was conducted by Lo and colleagues with a Lokomat robot, an exoskeleton device [15]. Lo and colleagues gave evidence that 4 individuals with Parkinson’s disease and symptoms of Frezing Of Gait (FOG), who received ten 30-minute sessions of exoskeleton robot-assisted gait training (Lokomat), showed a reduction in FOG both by self-report and clinician-rated scoring upon completion of training. Improvements were also observed in gait velocity, stride length, and coordination, although without statistical significance. Our study, which was conducted with an end-effector device, confirmed this work with a good statistical significance. The second recent study was performed by Picelli and colleagues [16]. Forty-one PD patients, randomly assigned to a robot-assisted gait training (RT) or physiotherapy (PT), showed a statistically significant improvement in favour of the robot therapy group performed by a Gait Trainer GT1 [16]. In this study, the authors compared the robot therapy with a standard physiotherapy. Studies of the effectiveness of treadmill training, to treat gait disturbance in people with Parkinson’s disease, have produced promising results compared with other physiotherapy approaches [29]. Our study is the first one that compares the robot with a treadmill device. In particular, our study was performed by the G-EO robot, an end-effector device designed explicitly to guide repetitive, rhythmic, bilateral lower extremity movements. This type of intense stereotyped somatosensory cueing and stimulation could help the functional recovery of the gait automatism and speed. At the beginning of the treatment, our patients exhibited a shorter step length, lower walking velocity and cadence, and larger step width, if compared to normal subjects, but the robot training helped individuals to obtain a good recovery of gait in term of mean velocity, step length and stride length. The same gain were also obtained with a treadmill PD training, but our sample does not show a significant statistical gain. The stance and swing phases did not show a change and the values at T0 were similar to those of a normal subject. In this pilot study, robot training produced a statistical improvement in term of walking capacity, walking speed and walking consistency, in accordance with thorough studies which have shown improvements in these measures following treadmill training [6]. One of the limitation of this study is related to the high cost of the robot but, on the other side, the robot is easy to use and needs one operator only for both the preparation of the patient and during its use. On the other hand, the treadmill device is currently present in various rehabilitative centres and it is normally used to treat PD patients with some improvement [30]. Our preliminary results, though, showed how this robot made an important gain, in gait recovery, with an important patient’s safety. The gain product with our end-effector robot is in accordance with a recent meta-analysis performed by Mehrholz, that analyses the walking recovery after cerebrovascular disease. The results of this analysis highlighted that the recovery may depend on the types of the training devices (end effector or exoskeleton) and, although there is an absence of a direct empirical comparison with electromechanical-assisted gait training devices, the meta-analysis results showed a superior effect in support of end-effectors treatment [9].

## Conclusions

The statistical improvement in a low number of people treated with robot device was also very interesting. In order to better investigate the result of this type of task-specific exercise training on gait kinetics and kinematics, any future study should include a larger number of subjects, as well as identifying underlying central and peripheral neuromuscular mechanisms with a long term follow-up.

## Competing interests

The authors declare that they have no competing interests.

## Authors’ contributions

All authors contributed to the design of the study, to draft and review the manuscript. AA, VC, MG contributed to data analysis. PS, MF, MFD and FS contributed in the supervision of the research study. All authors read and approved the final manuscript.

## Pre-publication history

The pre-publication history for this paper can be accessed here:

http://www.biomedcentral.com/1471-2377/13/50/prepub
